# Ablation of NLRP3 inflammasome rewires MDSC function and promotes tumor regression

**DOI:** 10.3389/fimmu.2022.889075

**Published:** 2022-08-10

**Authors:** Iosif Papafragkos, Maria Grigoriou, Louis Boon, Andreas Kloetgen, Aikaterini Hatzioannou, Panayotis Verginis

**Affiliations:** ^1^ Laboratory of Immune Regulation and Tolerance, Division of Basic Sciences, University of Crete Medical School, Heraklion, Greece; ^2^ Institute of Molecular Biology and Biotechnology, Foundation for Research and Technology, Heraklion, Greece; ^3^ Center of Clinical, Experimental Surgery and Translational Research, Biomedical Research Foundation Academy of Athens, Athens, Greece; ^4^ JJP Biologics, Warsaw, Poland; ^5^ Department of Computational Biology of Infection Research, Helmholtz Centre for Infection Research, Braunschweig, Germany; ^6^ Institute for Clinical Chemistry and Laboratory Medicine, Faculty of Medicine, Technische Universität Dresden, Dresden, Germany

**Keywords:** myeloid-derived suppressor cells (MDSCs), inflammasome, cancer immunotherapy, tumor immunity, tumor resistance

## Abstract

Myeloid-derived suppressor cells (MDSCs) are myeloid precursors that exert potent immunosuppressive properties in cancer. Despite the extensive knowledge on mechanisms implicated in mobilization, recruitment, and function of MDSCs, their therapeutic targeting remains an unmet need in cancer immunotherapy, suggesting that unappreciated mechanisms of MDSC-mediated suppression exist. Herein, we demonstrate an important role of NLRP3 inflammasome in the functional properties of MDSCs in tumor-bearing hosts. Specifically, *Nlrp3*-deficient mice exhibited reduced tumor growth compared to wild-type animals and induction of robust anti-tumor immunity, accompanied by re-wiring of the MDSC compartment. Interestingly, both monocytic (M-MDSCs) and granulocytic (G-MDSCs) subsets from *Nlrp3^-/-^* mice displayed impaired suppressive activity and demonstrated significant transcriptomic alterations supporting the loss-of-function and associated with metabolic re-programming. Finally, therapeutic targeting of NLRP3 inhibited tumor development and re-programmed the MDSC compartment. These findings propose that targeting NLRP3 in MDSCs could overcome tumor-induced tolerance and may provide new checkpoints of cancer immunotherapy.

## Introduction

The advent of immune checkpoint inhibitors (ICI) has revolutionized cancer immunotherapy. However, despite the enormous success, a significant proportion of patients do not respond (1), while responses are frequently accompanied by life-threatening autoimmune-related adverse events (irAEs) (2). Mounting evidence suggests that tumoral resistance and development of irAEs are dependent on the immunosuppressive nature of the tumor microenvironment (TME). It is therefore of paramount importance to delineate unappreciated mechanisms of resistance in order to design novel treatments aiming to confer robust and durable anti-tumor immunity. Accomplishment of this goal has been hampered by the multiple and complex immune suppressive networks operating during tumor development promoting tumor immune evasion (3). Myeloid-derived suppressor cells (MDSCs) are bone marrow (BM) progenitors of dendritic cells (DCs), macrophages, and neutrophils, composed by monocytic (M-MDSCs) and granulocytic (G-MDSCs) subsets (4). MDSCs constitute a major component of the tumor-induced immunosuppressive circuit since they are significantly enriched in the periphery and the TME of patients with solid tumors and hematologic malignancies (5) while MDSC presence is associated with poor prognosis as well as metastasis and is also linked to resistance to chemotherapy and immunotherapy (6, 7). Multiple mechanisms have been attributed to MDSC-mediated inhibition of anti-tumor immune responses, ranging from secretion of immunosuppressive mediators to direct cell-to-cell contact (6, 8). In preclinical models, targeting of such mechanism has generated promising results by promoting tumor regression and development of potent anti-tumor immunity. For example, targeting of autophagy pathway in M-MDSCs promoted the antigen-presenting properties of these cells and enhanced the anti-tumor immunity in a mouse model of melanoma (9). Furthermore, treatment with all-trans retinoic acid (ATRA) induced the differentiation of M-MDSCs into macrophages and DCs and killed G-MDSCs in both mice and humans (10–12). In addition, treatment of mice with fatty acid transporter 2 (FATP2) (13) or cyclooxygenase 2 (COX2) (14, 15) has been shown to interfere with MDSC expansion and to significantly attenuate tumor development. Finally, targeting of protein kinase R-like ER kinase (PERK) pathway induced the maturation of M-MDSCs and attenuated their function (16). However, the clinical translation of these findings remains in its infancy. Therefore, shedding light into mechanisms that mediate expansion and activation as well as arrest of differentiation of MDSCs may facilitate the design of new therapeutic target for immunotherapy in solid tumors and hematologic malignancies.

Chronic inflammation constitutes a hallmark of cancer. Inflammasomes and their effectors such as IL-1β and IL-18 significantly contribute to establishment of inflammation, while the TME is enriched in damage-associated molecular patterns (DAMPs) that have been shown to drive inflammasome activation in both immune and cancer cells. Among the best-studied inflammasomes, the NOD-like receptor family, pyrin domain containing-3 protein (NLRP3), has shown to be activated by DAMPs, followed by assembly of the NLRP3 complex and activation of caspase-1 in order to promote maturation of IL-1β and IL-18 cytokines (17, 18). Alternatively, sensing of cytoplasmic lipopolysaccharide (LPS) or Gram-negative bacteria induce inflammasome activation in a non-canonical manner, involving activation of caspase-11 upon type I IFN signaling, which, in turn, promotes IL-1β maturation and release through activation of the NLRP3/caspase-1 pathway (19). Although presence of IL-1β has been closely linked to tumor progression and metastasis in various types of cancer (20), the role of NLRP3 inflammasome activation remains controversial, suggesting that other functional roles of inflammasome, beyond secretion of pro-inflammatory mediators, may exist. Importantly, the impact of activation of NLRP3 in cancer cells versus the host cells during tumor immune surveillance remain ill defined. Considering that accumulating evidence proposes an important role of NLRP3 in chemotherapy success through induction of anti-tumor immunity (21, 22), while other studies highlight that activation of NLRP3 inflammasome impedes the effectiveness of ICI immunotherapy (23), it is necessary to unravel the molecular mechanism *via* which the inflammasome pathway imprints on anti-tumor immunity and effectiveness of immunotherapy.

Herein, we demonstrate that *Nlrp3* deficiency led to diminished tumor development, which was accompanied by a robust anti-tumor immunity and re-arrangement of the MDSC compartment. Both M-MDSCs and G-MDSCs from tumor-inoculated *Nlrp3^-/-^* mice lost their ability to suppress T-cell activation and proliferation and demonstrated an extensive transcriptomic reprogramming enriched in inflammatory and metabolic pathways. Notably, therapeutic inhibition of inflammasome significantly decreased tumor development and re-arranged the MDSC subsets, mirroring the effect described in *Nlrp3^-/-^* animals. Overall, uncovering of mechanisms that mediate tumor immune evasion may facilitate the development of new therapeutic opportunities for cancer patients.

## Methods

### TCGA survival analysis

Survival and gene expression information were downloaded from the TCGA data portal (https://portal.gdc.cancer.gov/) for SKCM (skin cutaneous melanoma) and LUSC (lung squamous cell carcinoma) datasets. We specifically downloaded preprocessed expression data using the quantile-normalized FPKM values. Next, patients were either stratified by expression of NLRP3 or the NLRP3-inflammasome-related gene set as defined by Ju et al. (24). For the NLRP3 inflammasome gene set, we defined a module score inspired by Seurat single-cell analysis (25), using each patient’s average expression of all NLRP3 inflammasome gene-set genes subtracted by the average expression of all genes. For survival analysis, we used the bottom and top 33% of patients with either NLRP3 expression or NLRP3 inflammasome module score. Statistical evaluation and Kaplan–Meier plot representation were performed with the R package survminer version 0.4.9, using the log-rank test for *p*-value estimation and 95% confidence interval of patient survival.

### Animals

C57BL/6J mice were purchased from the Jackson Laboratory; *Nlrp3*-deficient (*Nlrp3^-/-^*) mice (on a C57BL/6 background) were kindly provided by Jürg Tschopp (Department of Biochemistry, Center of Immunity and Infection, University of Lausanne, Switzerland) (26). Disruption of the mouse *Nlrp3* gene was based on the insertion of an EGFP cassette, which was accompanied by SV40 poly(A) tail, fused in frame with the ATG of exon 2. A PGK-neo selection cassette was also inserted in intron 2, which was flanked by two loxP sites and was deleted by the backcrossing of the mice, with the targeting vector, with a Cre-expressing strain (C57BL/6) resulting in a *Nlrp3^-/-^* mouse on a C57BL/6 background. Foxp3^EGFP^.KI mice (on a C57BL/6 background) were kindly provided by Alexander Rudensky (Department of Immunology, Memorial Sloan-Kettering Cancer Center, New York, USA).

All mice were maintained in the animal facility of the Biomedical Research Foundation of the Academy of Athens [BRFAA] and Institute of Molecular Biology and Biotechnology Institute [IMBB]. All procedures were in accordance with institutional guidelines and were approved by the Institutional Committee of Protocol Evaluation of the BRFAA and the Institutional Committee of Protocol Evaluation of the IMBB together with the Directorates of Agricultural Economy and Veterinary, Region of Crete, Greece (14/10/2020 Heraklion, Greece, protocol 234446). Unless indicated otherwise, all experiments used sex- and age-matched mice aged between 6 and 12 weeks.

### PCR Genotyping

*Nlrp3*-deficient mice were screened by PCR genotyping on tail genomic DNA using the following primers: 5’GCTCAGGACATACGTCTGGA3’ (forward in intron 1) and 5’TGAGGTCCACATCTTCAAGG3’ (reverse in exon 2). *Nlrp3^+/+^* (wild type) mice gave a product of 327 base pairs (bp), whereas *Nlrp3^-/-^* mice did not give any PCR product. The program used for the PCR genotyping is as follows: 94°C (3 min), 30 × [94°C (30 s), 58°C (30 s), 72°C (1 min)], and 72°C (5 min).

### Cell lines and primary cell culture

The murine melanoma cancer cell line B16.F10 and the murine Lewis Lung carcinoma (LLC) cell line that were used for the solid tumor induction models were kindly provided by A. Eliopoulos (Medical School, National and Kapodistrian University of Athens, Athens, Greece) and were negative for *Mycoplasma* spp., tested by PCR.

B16.F10 and LLC cancer cells were cultured at 37°C under 5% CO_2_ in RPMI-1640 (GlutaMAX™, Gibco, #61870) medium supplemented with 10% heat-inactivated fetal bovine serum (FBS, Gibco, #10270), 100 U/ml penicillin–streptomycin (10,000 U/ml, Gibco, #15140), and 50 μM 2-mercaptoethanol (50 mM, Gibco, #31350). Cells were split when they were 90%–100% confluent. All experiments were performed with early passage (p2–3) cells.

Splenocytes and sorted MDSCs were obtained as described below. Mouse splenocytes and MDSCs were grown in RPMI-1640 culture medium containing 10% heat-inactivated FBS, 100 U/ml penicillin–streptomycin, and 50 μM 2-mercaptoethanol. The stimuli at the cultures were added where indicated, as mentioned below.

### Solid tumor induction and *in vivo* immunotherapy administration protocols

The transplantation of solid tumors in the tumor models was performed as described previously (27). Briefly, mice were implanted subcutaneously, at the back, with 3 × 10^5^ B16.F10 melanoma or LLC cells (viability assessed by Trypan blue exclusion). Tumor volume was monitored during the days indicated in the legends of corresponding curves. The tumor growth was monitored by measurement of two perpendicular diameters of palpable tumors every day by a caliper and was calculated using the equation $\frac{\left(\begin{array}{c} length\;\times \;{\text{width}}^{2} \end{array}\right)}{2}$ . Mice were sacrificed and analysis was performed 15 days after tumor induction or as indicated each time. Mice with tumors larger than 1,100 mm^3^ were euthanized. Mice that manifested tumor ulceration were excluded for the experimental processes. At the endpoint of each experiment, the tumor weight was also determined.

For the application of the combinational therapy protocol, each mouse was treated with anti-CTLA-4 Ab (clone 4F10, Bioceros LB) at 100 μg per 100-μl dose and anti-PD-1 Ab (clone RMP1–14, Bioceros LB) at 200 μg per 100-μl dose intraperitoneally (i.p.) every 3 days after tumor implantation, whereas NLRP3 inhibitor (MCC950, Sigma-Aldrich, #5.38120.0001) was administered at 10 mg/kg dose to each mouse by i.p. injection every other day. Control mouse cohort was administered PBS on the same days.

### Tissue dissociation and sample preparation

For the analysis of tumor-infiltrating lymphocytes (TILs), single-cell suspensions were generated by dissecting and dissociating tumor tissue in the presence of collagenase D (1 mg ml^−1^, Roche) and DNase I (0.25 mg ml^−1^, Sigma), diluted in RPMI-1640 medium (Gibco), for 45 min at 37°C and then were homogenized and strained passing through a 40-μm pore size cell strainer (BD Falcon). For the analysis and isolation of different immune populations, single-cell suspensions from spleen and lymph nodes (LNs) were prepared by homogenization of the tissue and passing through a 40-μm pore size cell strainer. Isolated femoral and tibial bones from the hindlimbs were flushed with ice-cold 5% FBS in PBS for BM single-cell suspensions. Single-cell suspensions from spleen and BM were prepared after erythrocyte lysis with red blood cell lysis buffer.

### Flow cytometry, cell sorting, and quantification

For extracellular marker staining, single-cell suspensions from TILs, spleen, LNs, or BM were incubated for 20 min at 4°C with the following anti-mouse conjugated antibodies: CD45-PerCP/Cy5.5 (BioLegend, clone 30-F11, #103132, diluted 1:200), CD11c-PE/Cy7 (BioLegend, clone N418, #117318, diluted 1:200), CD11b-Brialliant Violet 510 (BioLegend, clone M1/70, #101263, diluted 1:200), CD11b-PE (BD Pharmingen, clone M1/70, #553311, diluted 1:200), Gr1-Pacific Blue (BioLegend, clone RB6-8C5, #108430, diluted 1:200), Gr1-Brilliant Violet 421 (BioLegend, clone RB6-8C5, #108434, diluted 1:200), Gr1-PE (eBioscience, clone RB6-8C5, #12-5931-82, diluted 1:200), Ly6G-PE (BioLegend, clone 1A8, #127608, diluted 1:200), Ly6C-Brilliant Violet 421 (BioLegend, clone RB6-8C5, #108430, diluted 1:200), CD8a-PE/Cy7 (BioLegend, clone 53-6.7, #100722, diluted 1:200), CD8a-PE (BioLegend, clone 53-6.7, #100708, diluted 1:200), CD4-PE (BioLegend, clone RM4-4, #116006, diluted 1:200), NK-1.1-APC (BioLegend, clone PK136, #108710, diluted 1:200), CD16/32-PE (BioLegend, clone 93, #101308, diluted 1:200), CD16/32-PerCP/Cy5.5 (BioLegend, clone 93, #101323, diluted 1:200), TER-119/Erythroid Cells-PE (BioLegend, clone TER-119, #116208, diluted 1:200), CD45R/B220-PE (BioLegend, clone RA3-6B2, #103208, diluted 1:200), Ly-6A/E (Sca-1)-APC (BioLegend, clone E13-161.7, #122512, diluted 1:200), CD117 (c-kit)-PE/Cy7 (BioLegend, clone 2B8, #105813, diluted 1:200), CD34-Brilliant Violet 421 (BioLegend, clone MEC14.7, #119321, diluted 1:200), CD44-PerCP/Cy5.5 (BioLegend, clone IM7, #103032, diluted 1:200), and CD25-PE (BioLegend, clone 3C7, #101904, diluted 1:200). Dead cells in cultured splenocytes were stained by the addition of 7-AAD Viability Staining Solution (BioLegend, #420404). For NLRP3-APC (R&D Systems, clone 768319, #IC7578A, diluted 1:25) and IL-1β-FITC (R&D Systems, clone 166931, #IC4013F, diluted 1:50) intracellular staining, cells were stained for the extracellular markers, and then permeabilized and stained using the intracellular Fixation & Permeabilization buffer set (eBioscience) according to the vendor’s instructions. Rat IgG1 kappa Isotype Control-APC (eBioscience, clone eBRG1, #17-4301-81, diluted 1:100) and Rat IgG2b kappa Isotype Control-FITC (eBioscience, clone eB149/10H5, # 11-4031-82, diluted 1:100) were used as controls for NLRP3 and IL-1β, respectively. For IFN-γ intracellular staining, tumor cells were incubated with 50 ng ml^−1^ of phorbol 12-myristate 13-acetate (PMA, Sigma-Aldrich), 2 μg ml^−1^ of ionomycin (Sigma-Aldrich), and brefeldin (1/1,000; Becton Dickinson Biosciences) for 4 h at 37°C and 5% CO_2_, stained for extracellular markers, and fixed and stained for IFN-γ (BioLegend, clone XMG1.2, #505808, diluted 1:50) or Rat IgG2a kappa Isotype Control-PE (eBioscience, clone eBR2a, #12-4321-81, diluted 1:50) using Foxp3 Transcription Factor Staining Buffer Set (eBioscience) according to the manufacturer’s instructions. All samples were analyzed using FACS ARIA III (BD Biosciences) and FACS Canto II (BD Biosciences). Flow cytometry data were analyzed with FlowJo v.8.7 and 10.8.1 software. MDSCs were sorted on a FACS ARIA III (BD Biosciences) and the BD FACSDIVA v8.0.1 software (BD Biosciences). Cell purity was above 95%.

Calculation of TIL numbers per gram of tumor tissue was performed, by flow cytometry, upon tumor tissue isolation, weighing, digestion, and suspension in a 0.1 g/100 μl volume of 5% FBS in PBS prior to staining.

### Inflammasome activation assays

Splenocytes as well as sorted splenic MDSCs from naïve and tumor-bearing mice were isolated as previously described. We seeded 5 × 10^5^ splenocytes or 1 × 10^5^ MDSCs per well in 96-well flat-bottom and 96-well round-bottom plates, respectively, and stimulated them for 24 h with 1 μg/ml LPS from *Escherichia coli* O55:B5 (Sigma, L2880). The next day, 1 h before the ending of the stimulation time, the culture medium was supplemented with 5 mM adenosine 5’-triphosphate disodium salt hydrate (ATP) inflammasome activator (Jena Bioscience, NU-1010-1G). Supernatants were removed and analyzed using Mouse IL-1 beta/IL-1F2 Quantikine ELISA Kit (R&D Systems, MLB00C), according to the manufacturer’s instructions, and cultured cells were prepared for staining of extracellular and intracellular markers, as described above.

### MCC950-mediated inflammasome inhibition assays

Total splenocytes from naïve mice were seeded 5 × 10^5^ cells per well in 96-well flat-bottom plates and stimulated for 24 h with 1 μg/ml LPS (Sigma, L2880). The following day, in the medium from the overnight culture was added water for injection (control) or MCC950 NLRP3 inhibitor (2 μM) (Sigma-Aldrich, 5381200001) for the indicated time points. One hour before the end of priming, cells were stimulated with ATP inflammasome activator (Jena Bioscience, NU-1010-1G). Supernatants were removed and used for mouse IL-1β ELISA and cultured splenocytes were prepared for 7-AAD viability staining, as described above.

### *In vitro* suppression assay

For the suppression assay of MDSC subsets, CD4^+^Foxp3^-^ effector T cells (Teff) were sorted from the LNs of naïve Foxp3^EGFP^.KI mice, as previously described, and stained with the division-tracking dye CellTrace Violet (CTV, Invitrogen, #C34557) according to the manufacturer’s protocol. A total of 75 × 10^3^ labeled Teff cells were then seeded in 96-well round-bottom plate in each well. M-MDSC (CD11b^high^Ly6C^+^Ly6G^–^) and G-MDSC (CD11b^high^Ly6C^–^Ly6G^+^) subsets sorted from the spleens of WT or *Nlrp3^-/-^* B16.F10 inoculated mice were added at the culture, at a ratio Teff/M-MDSCs 1:1 and Teff/G-MDSCs 3:1. Then, Dynabeads mouse T-activator conjugated with monoclonal antibody (mAb) to the invariant signaling protein CD3 plus mAb to CD28 (Gibco, #11456D) were supplemented into culture, at a ratio of one bead per one Teff cell. Cells were cultured in DMEM (Gibco, #11965) supplemented with 10% heat-inactivated FBS, 100 U/ml penicillin–streptomycin, and 50 μM 2-mercaptoethanol. As positive and negative controls, we used Teff cells cultured with or without anti-CD3/anti-CD28 activation beads, respectively. The plate was incubated at 37°C under 5% CO_2_ for 64 h and then cultured cells were prepared for staining of extracellular markers, as described above, for the determination of proliferation and activation of Teff cells with flow cytometry analysis.

### Enzyme-linked immunosorbent assay

Tumor homogenates were generated in PBS that was supplemented with a cocktail of protease inhibitors (Roche) using a pestle inside an Eppendorf. The conditioned media of cultured splenocytes and MDSCs from *in vitro* cultures were also collected. The homogenates and cell culture supernatants were centrifuged and were assayed for mouse IL-1β using the Mouse IL-1 beta/IL-1F2 Quantikine ELISA Kit (R&D Systems, MLB00C), according to the manufacturer’s instructions.

### RNA-seq library preparation

M-MDSCs and G-MDSCs were sorted from spleens of B16.F10 melanoma-bearing WT and *Nrlp3^-/-^* mice, and total RNA was extracted using the Macherey-Nagel NucleoSpin RNA kit as described by the manufacturer’s protocol (NucleoSpin^®^ RNA). Each RNA sample was representative of one mouse.

NGS libraries were generated using 300 ng of total RNA as input on average with the QuantSeq 3′ mRNA-Seq Library Prep Kit FWD for Illumina kit from Lexogen according to manufacturer’s protocol, using 15 or 17 cycles of amplification. Libraries were sequenced on Illumina Nextseq 500 on 1 × 75 High flowcell.

### RNA sequencing pipeline

Fastq files were downloaded from Illumina-BaseSpace and mapped to mm10 genes (iGenomes UCSC/mm10) using hisat2 version 2.1.0 (–score-min L 0,-0.5) (28). Gene counts were computed with htseq-count (-s yes, version 0.11.2) (29).

Further processing was performed with the R Bioconductor (Bioconductor) package edgeR v.3.14.0 (edgeR). Reads were normalized for intra- and inter-sample variances using the functions “calcNormFactors” and “estimateTagwiseDisp”, and further by the gene length as per Ensembl V103 Genes annotations, resulting in fragments per kilobase per million (FPKM) for each gene. Differential gene expression analysis was performed as previously described (30). Genes with FDR< 0.05 and fold change |FC| > 1.5 were considered statistically significant. Heatmaps and boxplots were created in R with an in-house developed script that is based on the ggplot package.

### Enrichment analysis

Significant differentially expressed genes (DEGs) were used for gene ontology (GO) analysis using the g:Profiler web-server. Gene set enrichment analysis (GSEA) was also performed in order to reveal enriched signatures in our gene sets based on the Molecular Signatures Database (MSigDB) v7.4. Gene sets were ranked by taking the –log10 transform of the *p*-value multiplied by the FC. Significantly upregulated genes were at the top and significantly downregulated genes were at the bottom of the ranked list. GSEA pre-ranked analysis was then performed using the remapped Mouse Gene Symbol dataset and collapsing probe sets while keeping only the max probe value. The rest of the parameters were left to default. Enrichment was considered significant FDR (*q*-value)<5%.

### Data analysis and statistics

Data are presented as mean ± S.D., as bar graphs represent the mean and standard deviation (SD) between biologically independent mouse samples or technical repeats, as indicated each time. For statistical analysis, all data were analyzed using Prism 8 (GraphPad Software, Inc., La Jolla, USA). Data were analyzed using the two-tailed, parametric, unpaired Student’s t test or the two-tailed, nonparametric Mann–Whitney test, as appropriate after testing for normality of the values with the *F* test, with 95% confidence intervals. For multiple-group comparisons, the one-way ANOVA test and Tukey’s multiple comparison test were performed. Kaplan–Meier statistics were done with the log(rank) (Mantel–Cox) test. The *p-*value of<0.05 was considered to be statistically significant for each dataset.

## Results

### NLRP3 inflammasome pathway genes are associated with survival in melanoma and lung cancer patients

**Figure 1:**
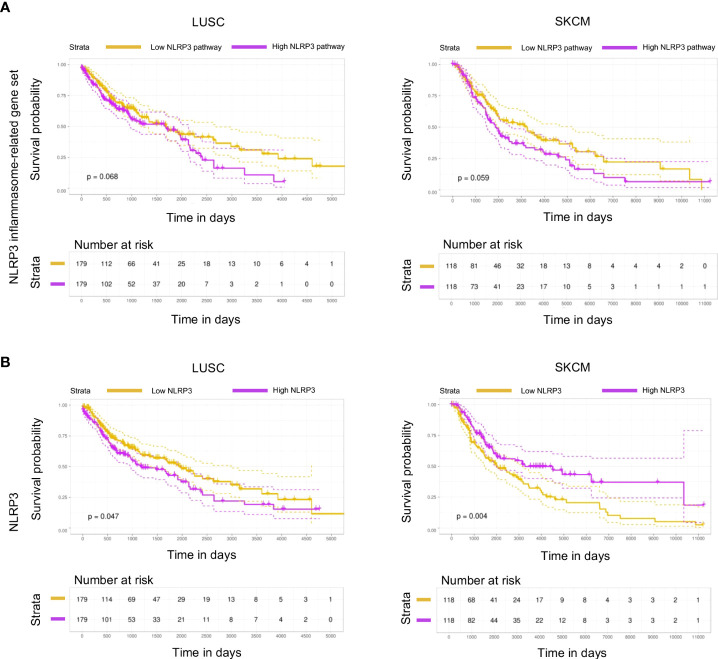
NLRP3 expression is associated with survival in melanoma and lung cancer patients. **(A,B)** Kaplan–Meier plot with patients-at-risk table showing overall survival of TCGA datasets LUSC (lung squamous cell carcinoma; left) and SKCM (skin cutaneous melanoma; right), with patients stratified by NLRP3 inflammasome-related gene set **(A)** and NLRP3 **(B)** expression using top and bottom 33% of stratified patients. Gene-set stratification is based on the median expression of all 30 containing genes. Dotted lines represent 95% confidence intervals. Statistical evaluation was performed using the log-rank test.

Despite the established role of inflammasome pathway in the orchestration of an inflammatory response, through release of pro-inflammatory cytokines IL-1β and IL-18, its role in cancer development, progression, and immunotherapy response remains contradictive. To investigate the impact of NLRP3 inflammasome in the progression of LUSC and SKCM, Kaplan–Meier survival analysis was performed based on high and low NLRP3 gene expression or NLRP3 pathway gene-set expression [median expression of the 30 pathway genes as defined by Ju et al. (24), **Supplementary Figure 1A**], using the top/bottom 33% of samples with the highest/lowest expression, respectively. Patients with the lowest expression of both NLRP3 gene and its pathway gene set were found to display prolonged survival of LUSC patients (*p* = 0.068 and *p* = 0.047, respectively, **Figures 1A, B**, left panels). In accordance, low expression of the NLRP3 pathway gene set was also associated with prolonged survival of SKCM patients (*p* = 0.059, **Figure 1A**, right upper panel), whereas in these patients, low expression of NLRP3 gene was associated with increased morbidity rate (*p* = 0.004, **Figure 1B**, right lower panel). In summary, these data indicate that differential expression of NLRP3 pathway genes is associated with survival probability of cancer patients and extend the findings that NLRP3 inflammasome may have pro- and anti-tumorigenic roles.

### *Nlrp3*-deficient mice exhibit attenuated tumor development and re-arranged MDSC compartment

To examine the functional importance of NLRP3 pathway during tumor development, we utilized the *Nlrp3*-deficient mice (*Nlrp3^-/-^*), in which the *Nlrp3* gene has been disrupted by the insertion of an EGFP cassette (**Supplementary Figure 2A**). Effective inactivation of NLRP3 inflammasome was confirmed by the absence of IL-1β in culture supernatants of LPS/ATP-stimulated splenocytes from *Nlrp3^-/-^* animals compared to WT mice (**Supplementary Figure 2B**). Furthermore, *Nlrp3^-/-^* animals did not exhibit significant alterations in the composition of lymphoid (**Supplementary Figures 2C, D**) and myeloid compartments (**Supplementary Figures 2E, F**) compared to WT mice at the steady state, suggesting that inactivation of *Nlrp3* gene does not disturb the immune homeostasis.

**Figure 2:**
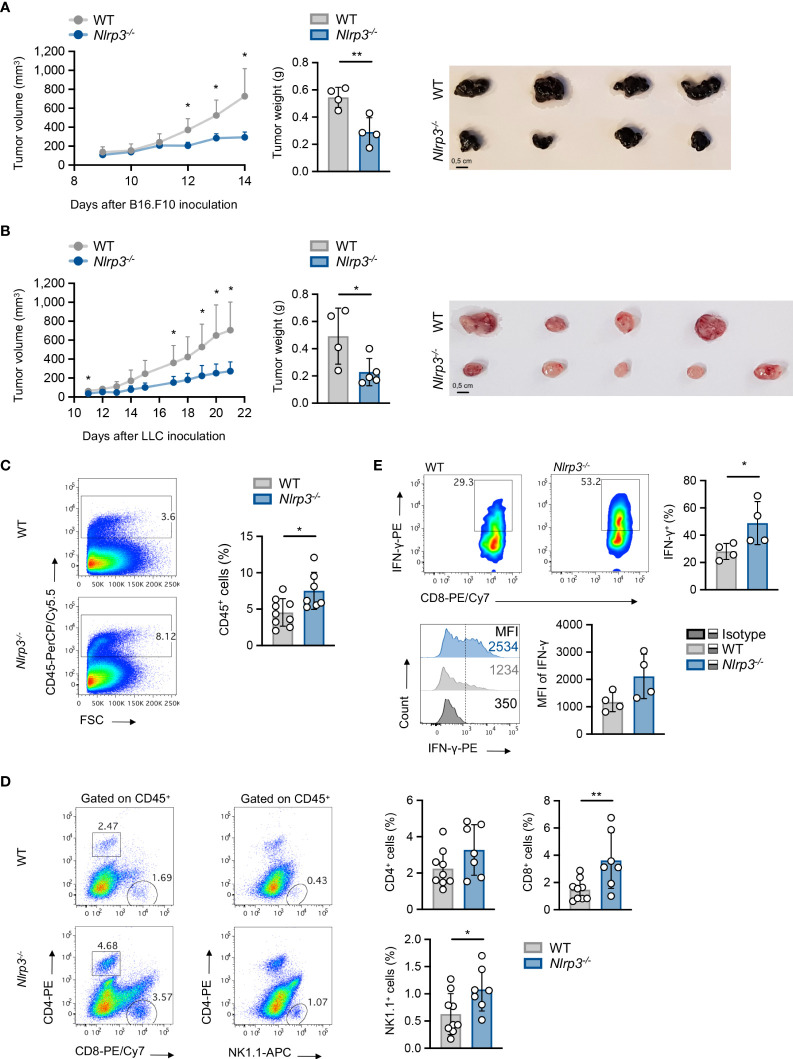
Nlrp3 deficiency promotes tumor regression and anti-tumor immunity. **(A)** Tumor volume curve of WT (*n* = 4) and *Nlrp3^-/-^* (*n* = 4) mice 9–14 days after B16.F10 inoculation, tumor weight on day 14, and representative image of excised melanoma tumors. **(B)** Tumor volume curve of WT (*n* = 4) and *Nlrp3^-/-^* (*n* = 5) mice 11–21 days after LLC inoculation, tumor weight on day 21, and representative image of excised LLC tumors. **(C)** Representative fluorescence-activated cell sorting (FACS) plots and percentages of intratumoral CD45^+^ cells of WT (*n* = 9) and *Nlrp3^-/-^* (*n* = 7) mice 15 days after B16.F10 inoculation. **(D)** Gating strategy and frequencies of intratumoral CD4^+^, CD8^+^, and NK1.1^+^ cells in CD45^+^ population of WT (*n* = 9) and *Nlrp3^-/-^* (*n* = 7) mice 15 days after B16.F10 inoculation. **(E)** Representative FACS plots and frequencies of CD8^+^ IFN-γ^+^ cells from CD45^+^ tumor-infiltrating cells of WT (*n* = 4) and Nlrp3^-/-^ (*n* = 4) mice 15 days after B16.F10 inoculation, and representative overlay and mean fluorescence intensity (MFI) of IFN-γ produced by CD8^+^ cells after *in vitro* activation. Data are shown as mean ( ± S.D.). Representative data from at least two independent experiments are shown. Statistical significance was obtained by unpaired Student’s *t*-test. Symbols: (*), *p* ≤ 0.05; (**), *p* ≤ 0.01, *n* = biologically independent mouse samples.

**Figure 3:**
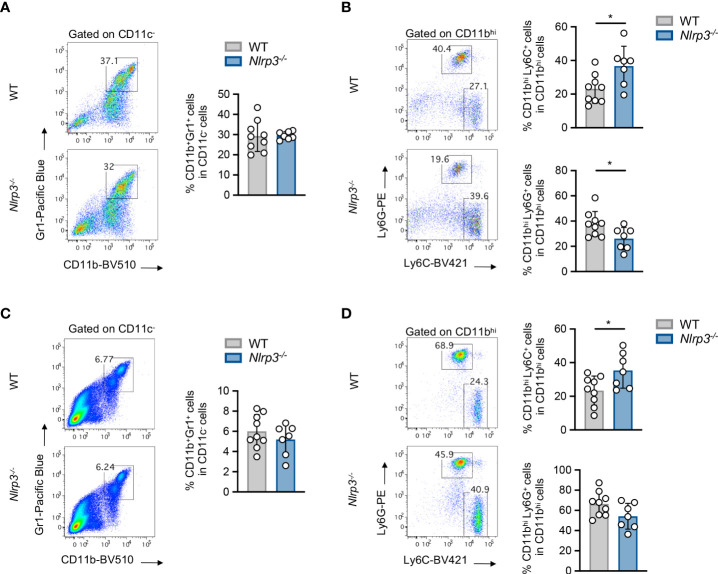
Nlrp3 deficiency re-arranges the MDSC compartment. **(A, B)** Representative FACS plots and frequencies of intratumoral MDSC population **(A)** and MDSC subpopulations **(B)**, M-MDSCs and G-MDSCs, of WT (*n* = 9) and *Nlrp3^-/-^* (*n* = 7) mice 15 days after B16.F10 inoculation. **(C, D)** Representative FACS plots and frequencies of spleen-infiltrating MDSC population **(C)** and MDSC subpopulations **(D)**, M-MDSCs and G-MDSCs, of WT (*n* = 9) and *Nlrp3^-/-^* (*n* = 7) mice on day 15 after B16.F10 inoculation. Data are shown as mean ( ± S.D.). Representative data from four independent experiments are shown. Statistical significance was obtained by unpaired Student’s *t*-test. Symbols: (*), *p* ≤ 0.05, *n* = biologically independent mouse samples.

Therefore, this model allows us to study the role of the NLRP3 pathway in host cells through implantation of NLRP3-sufficient tumor cell line. To this end, upon inoculation with B16.F10 melanoma cells, *Nlrp3^-/-^* mice exhibited significantly reduced tumor growth compared to WT mice, as assessed by the measurement of tumor volume and weight (**Figure 2A**). The tumor-suppressive effect of NLRP3 deficiency was not restricted only to the melanoma model, since inoculation with the LLC cell line demonstrated significantly decreased tumor growth in *Nlrp3^-/-^* animals (**Figure 2B**). Tumor regression in melanoma-bearing *Nlrp3^-/-^* mice was accompanied by significantly increased frequencies of tumor-infiltrating CD45^+^ leukocytes (**Figure 2C**), CD8^+^ lymphocytes, which exhibited elevated IFN-γ expression, and NK1.1^+^ cells, as well as increased frequencies of CD4^+^ T cells, compared to WT mice (**Figures 2D, E**). These findings were further confirmed upon extrapolation of cell subset frequencies to respective numbers per gram of tumor tissue (**Supplementary Figure 3A**). Interestingly, CD11c^–^CD11b^+^Gr1^+^ MDSCs frequencies were comparable between WT and *Nlrp3^-/-^* mice (**Figure 3A**), whereas assessment of MDSC subsets revealed an increased accumulation of CD11b^high^Ly6C^+^Ly6G^–^ M-MDSCs and markedly decreased levels of CD11b^high^Ly6C^–^Ly6G^+^ G-MDSCs into the tumor site of *Nlrp3^-/-^* mice (**Figure 3B**). The MDSC subset re-arrangement was also evident in the spleen of tumor-inoculated WT and *Nlrp3^-/-^* mice (**Figures 3C, D**). Overall, these findings indicate an important role of NLRP3 in the suppression of anti-tumor immune responses and tumor development, associated with a major rewiring of the MDSC compartment.

### Enhanced activation of NLRP3/pro-IL-1β axis in the myeloid compartment of tumor-bearing mice

**Figure 4:**
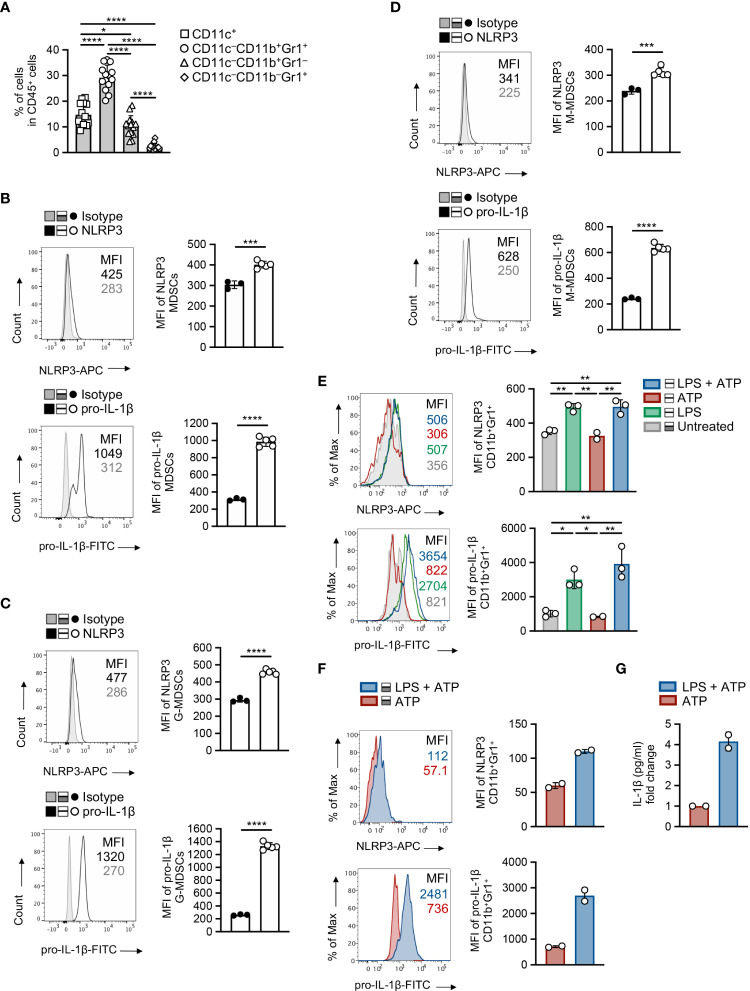
MDSCs exhibit increased activation of NLRP3 during tumor development. **(A)** Frequencies of intratumoral myeloid cell subsets in CD45^+^ population on day 15 of B16.F10 melanoma tumors (*n* = 14), determined by flow cytometric analysis. **(B)** Representative histogram overlays of NLRP3 and pro-IL-1β expression and plots of NLRP3 (*n* = 5) and pro-IL-1β (*n* = 5) MFI of spleen-infiltrating MDSCs, compared to isotype controls (*n* = 3), on day 15 after B16.F10 inoculation. **(C, D)** Representative histogram overlays of NLRP3 and pro-IL-1β expression and plots of NLRP3 (*n* = 5) and pro-IL-1β (*n* = 5) MFI of spleen-infiltrating G-MDSCs **(C)** and M-MDSCs **(D)**, compared to isotype controls (*n* = 3), as in **(B)**. **(E, F)** Representative histogram overlays of NLRP3 and pro-IL-1β expression and quantitative plots of MFI of NLRP3 and pro-IL-1β MFI with quantitative plots of NLRP3 and pro-IL-1β MFI in WT splenocytes from naïve mice (*n* = 3) gated on MDSCs **(E)** and sorted MDSCs from the spleens of tumor-bearing mice (*n* = 2) **(F)**, primed with LPS (1 μg/ml) and stimulated for NLRP3 activity by ATP (5 mM). **(G)** Quantification of IL-1β levels (pg ml^-1^) in the supernatants of LPS and/or ATP stimulated sorted MDSCs (*n* = 2), determined by ELISA (graph shows the fold change relative to ATP). Data from one experiment are shown **(F, G)**. Data are shown as mean ( ± S.D.). Representative data from at least two **(A–E)** independent experiments are shown. Statistical significance was obtained by unpaired Student’s *t*-test or one-way ANOVA with Tukey’s multiple comparison test (a, e). Symbols: (*), *p* ≤ 0.05; (**), (***), *p* ≤ 0.001, *p* ≤ 0.01; (****), *p* ≤ 0.0001, *n* = biologically independent mouse samples.

Based on the established expression of NLRP3 in the myeloid compartment (31) combined with the enhanced accumulation of MDSCs compared to other myeloid cell subsets in the TME (**Figure 4A** and **Supplementary Figure 4A**) and the extended re-arrangement of MDSC subsets in the tumor-inoculated *Nlrp3^-/-^* mice, we sought to determine the NLRP3 and pro-IL-1β expression in the MDSC population during tumor development. To this end, MDSCs demonstrated enhanced expression of NLRP3, which was accompanied by increased expression of pro-IL-1β, in spleen of melanoma-bearing animals, compared to isotype controls (**Figure 4B**). This was also confirmed to M-MDSC and G-MDSC subpopulations (**Figures 4C, D**), with the latter to express higher levels of both NLRP3 and pro-IL-1β (**Supplementary Figure 4B**).

Next, we asked whether NLRP3 inflammasome is functionally active in MDSCs, and to address this, we exposed them to cellular insults that include “priming” signal with a TLR agonist, such as LPS, and an activation stimulus, such as ATP. Flow cytometry analysis demonstrated robust expression of NLRP3 and pro-IL-1β in MDSCs from spleens of naïve mice (**Figure 4E**) as well as in highly pure MDSCs isolated from tumor-bearing mice (**Figure 4F**), and this is accompanied by increased levels of secreted IL-1β as determined in culture supernatants (**Figure 4G**). Overall, these data indicate that the NLRP3 inflammasome pathway is activated and functional in MDSCs during tumor development.

### Decreased frequencies of granulocyte-myeloid progenitors in the BM of *Nlrp3^-/-^* mice

MDSCs are BM-derived cells, and recent evidence highlights the importance of BM hematopoiesis and in particular of myeloid progenitors in shaping of anti-tumor immunity (32). Thus, we asked whether *Nlrp3^-/-^* animals present alterations of BM progenitors during tumor growth, which may affect the generation of MDSC subsets. To this end, flow cytometry analysis revealed that tumor-bearing *Nlrp3^-/-^* mice did not exhibit significant differences in frequencies of hematopoietic progenitors (LSKs; Lin^–^cKit^+^Sca1^+^) in their BM as compared to the tumor-bearing WT group (**Supplementary Figures 5A, B**). In line with this, assessment of total myeloid progenitor frequencies (MyPs; Lin^–^cKit^+^Sca1^–^) did not demonstrate any significant difference between *Nlrp3^-/-^* and WT mice (**Supplementary Figures 5C, D**) with granulocyte macrophage progenitors (GMPs; Lin^–^cKit^+^Sca1^–^CD16/32^+^CD34^+^) to be decreased in *Nlrp3^-/-^* mice while common myeloid progenitors (CMPs; Lin^–^cKit^+^Sca1^–^CD16/32^–^CD34^+^) were not affected (**Supplementary Figures 5C, E**). To conclude, these data suggest that *Nlrp3^-/-^* mice did not exhibit differences in the hematopoietic progenitors but granulocyte progenitors are decreased in tumor-bearing animals.

#### Transcriptomic re-programming of both M- and G-MDSCs in tumor-bearing *Nlrp3^-/-^* animals

To investigate the molecular mechanisms *via* which NLRP3 deficiency imprints on the re-wiring of MDSC subsets, highly pure M-MDSCs and G-MDSCs isolated from spleen of B16.F10-bearing *Nlrp3^-/-^* and WT mice were subjected to mRNA sequencing (mRNA-seq) and gene expression analysis. A combined principal component analysis (PCA) of all RNA-seq samples showed clustering by both factors, subpopulation and species. While PC1, explaining 46.08% of the total variance, separates G-MDSCs from M-MDSCs, PC2, explaining 15.84% of the total variance, differentiates pure deficient samples from control samples (**Supplementary Figure 6A**).

**Figure 5:**
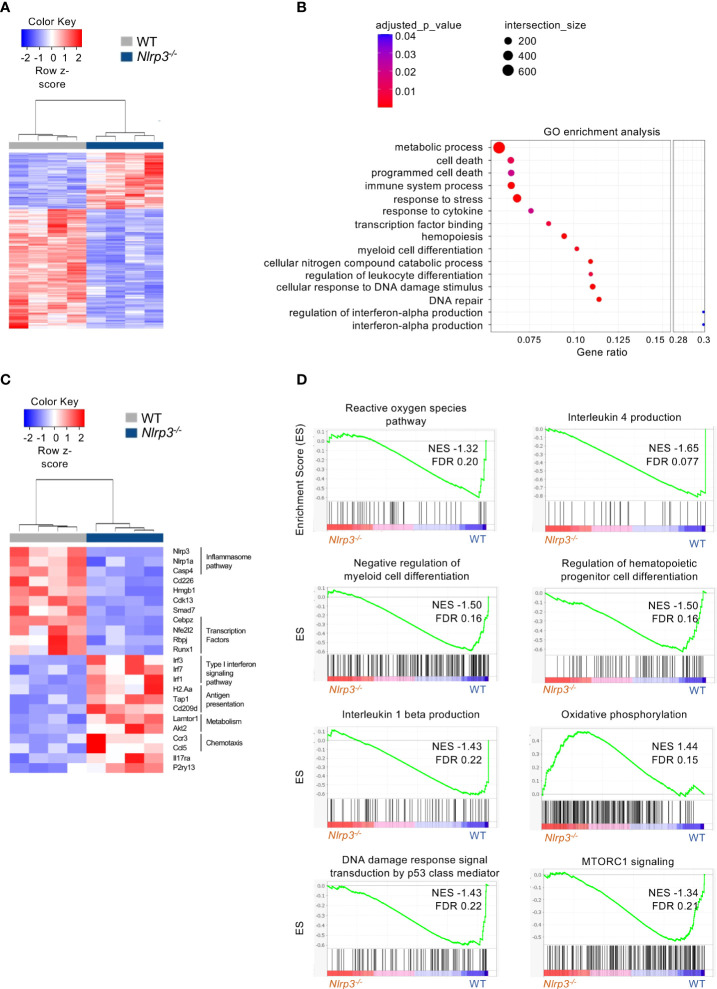
Extensive transcriptomic re-programming of *Nlrp3^-/-^* M-MDSCs. **(A)** Heatmap of DEGs from M-MDSCs from tumor-bearing *Nlrp3^-/-^* (*n* = 4) and WT (*n* = 4) mice. **(B)** Pathway analysis of DEGs from *Nlrp3^-/-^* vs. WT. **(C)** Heatmap of selected DEGs from M-MDSCs from tumor-bearing *Nlrp3^-/-^* and WT mice. **(D)** GSEA plot showing the enrichment of “Reactive oxygen species pathway” (NES −1.32, FDR 0.20), “Interleukin 4 production” (NES −1.65, FDR 0.077), “Negative regulation of myeloid cell differentiation” (NES −1.50, FDR 0.16), “Regulation of hematopoietic progenitor cell differentiation” (NES −1.50, FDR 0.16), “Interleukin 1-beta production” (NES −1.43, FDR 0.22), “Oxidative phosphorylation” (NES 1.44, FDR 0.15), “DNA damage response signal transduction by p53 class mediator” (NES −1.43, FDR 0.22), and “MTORC1 signaling” (NES −1.34, FDR 0.21) gene set.

Specifically, transcriptomic analysis in M-MDSCs revealed 992 DEGs (|FC| ≥ 1.5, FDR< 0.05) between *Nlrp3^-/-^* and WT melanoma-bearing animals (**Figure 5A**) with 318 and 674 genes to be up- and downregulated, respectively (**Supplementary Figure 6B**). Pathway analysis of DEGs performed on Gene Ontology terms demonstrated that M-MDSCs from *Nlrp3^-/-^* mice exhibited an inflammatory phenotype consisting of pathways involved in the interferon-alpha production, myeloid cell differentiation, and the response to DNA damage and repair pathways (**Figure 5B**). Importantly, transcription factors that have been closely linked to the suppressive activity of MDSCs (16, 33) (i.e., Cebpz, NF-E2-related factor 2) were downregulated in *Nlrp3-*deficient M-MDSCs (**Figure 5C**). Furthermore, genes associated with the antigen presentation process (*H2Aa*, *Tap1*, and *Cd209d*), as well as genes related to metabolic processes (*Lamtor1*, *Akt2*) were increased in M-MDSCs from *Nlrp3^-/-^* mice (**Figure 5C**). In support, GSEA, based on the Molecular Signatures Database (MSigDB) Hallmark and Gene Ontology gene set collections, revealed that *Nlrp3-*deficient M-MDSCs were metabolically re-programmed since they demonstrate enriched expression of transcripts related to the “oxidative phosphorylation” (NES 1.44, FDR 0.15) gene set, but were negatively correlated with “mTORC1 signaling” (NES −1.34, FDR 0.21) and “reactive oxygen species” (NES −1.32, FDR 0.20) (**Figure 5D**).

**Figure 6:**
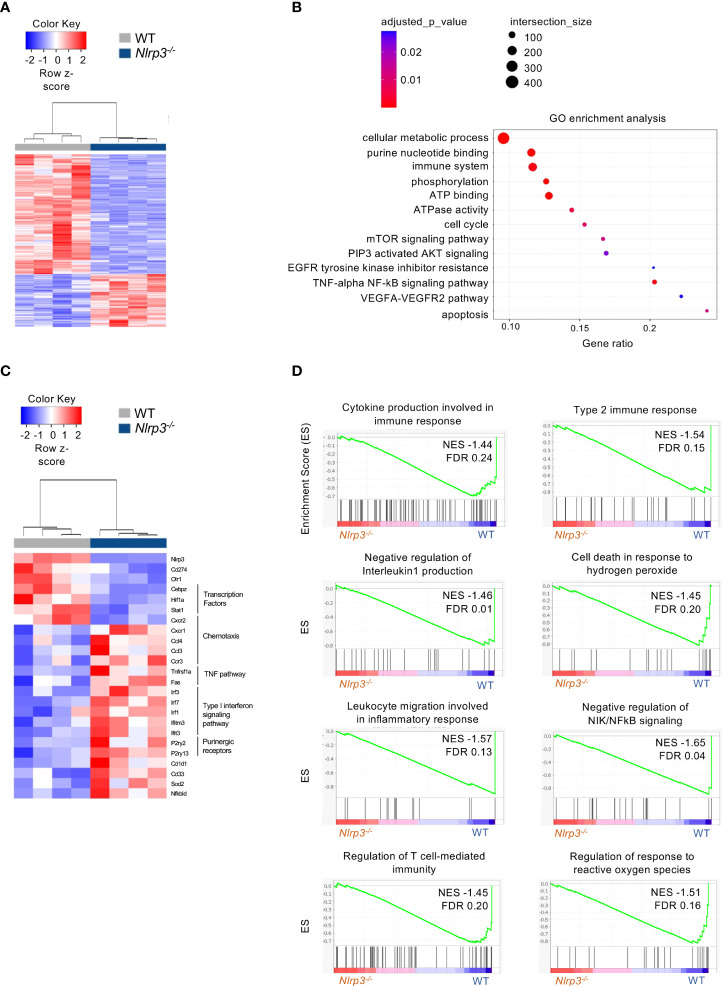
NLRP3 deficiency induces a transcriptional re-wiring to G-MDSCs. **(A)** Heatmap of DEGs from G-MDSCs from tumor-bearing *Nlrp3^-/-^* (*n* = 4) and WT (*n* = 4) mice. **(B)** Pathway analysis of DEGs from *Nlrp3^-/-^* vs. WT. **(C)** Heatmap of selected DEGs from G-MDSCs from tumor-bearing *Nlrp3^-/-^* and WT mice. **(D)** GSEA plot showing the enrichment of “Cytokine production involved in immune response" (NES −1.44, FDR 0.24), “Type 2 immune response” (NES −1.54, FDR 0.15), “Negative regulation of interleukin 1 production” (NES −1.46, FDR 0.01), “Cell death in response to hydrogen peroxide” (NES −1.45, FDR 0.20), “Leukocyte migration involved in inflammatory response” (NES −1.57, FDR 0.13), “Negative regulation of NIK/NFkB signaling” (NES −1.65, FDR 0.04), “Regulation of T cell-mediated immunity” (NES −1.45, FDR 0.20), and “Regulation of response to reactive oxygen species” (NES −1.51, FDR 0.16) gene set.

Regarding the G-MDSCs, differential expression analysis revealed 1,521 DEGs (|FC| > 1.5, FDR< 0.05) between tumor-bearing *Nlrp3-*deficient and WT mice (**Figure 6A**), with 466 and 1,055 genes to be up- and downregulated, respectively (**Supplementary Figure 6B**). Pathway analysis showed an enrichment in tumor necrosis factor (TNF)-alpha/NF-kB as well as the mTOR and AKT signaling pathways (**Figure 6B**), consistent with an inflammatory re-programming of *Nlrp3-*deficient G-MDSCs. Interestingly, expression of oxidized low-density lipoprotein receptor 1 gene (*Olr1*, also known as LOX-1), a signature gene in G-MDSCs (34), was downregulated in *Nlrp3-*deficient cells (**Figure 6C**). In addition, expression of *Cd274* gene (known as PDL1), closely linked to tumor immune evasion (35, 36), was downregulated in *Nlrp3^-/-^* G-MDSCs (**Figure 6C**). Notably, type I interferon genes were upregulated in G-MDSCs from *Nlrp3^-/-^* mice consistent with loss of suppressive function (32) (**Figure 6C**). Finally, GSEA revealed a metabolic rewiring in *Nlrp3-*deficient G-MDSCs as evident by the negative enrichment of the “regulation of response to reactive oxygen species” (NES −1.51, FDR 0.16) gene set (**Figure 6D**), which was further supported by the upregulation of superoxide dismutase 2 (*Sod2*) expression, an antioxidant enzyme crucial for mtROS scavenging (37), which is known to be highly expressed in mature neutrophils (38) (**Figure 6C**). Conclusively, these findings support an extensive transcriptomic re-programming of both M- and G-MDSCs in *Nlrp3^-/-^* tumor-inoculated animals, consistent with an inflammatory and less-suppressive phenotype.

### *Nlrp3*-deficient MDSC subsets demonstrated impaired suppressive activity

**Figure 7:**
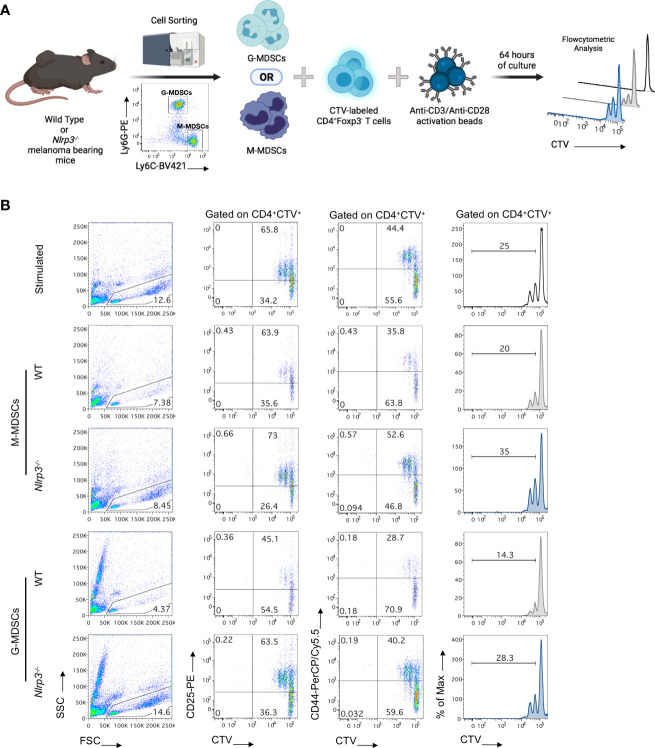
MDSC subsets from *Nlrp3^-/-^* mice exhibit an impaired suppressive function. **(A)** Schematic representation of the *in vitro* experimental setup for the evaluation of the suppressive ability of MDSC subsets. M-MDSCs and G-MDSCs were sorted from the spleen of WT (*n* = 2) and *Nlrp3^-/-^* (*n* = 2) melanoma-bearing mice and were co-cultured, at different ratios, with CellTrace Violet (CTV)-labeled T effectors (CD4^+^Foxp3^–^) cells, isolated from the lymph nodes (LNs) of Foxp3^GFP^ naïve mice and activated by anti-CD3/anti-CD28 monoclonal antibodies. Suppressive activity of MDSC subsets was estimated 64 h later by flow cytometry. **(B)** Representative FACS plots showing the percentages of lymphocytes according to FSC and SSC, frequencies of CD4^+^CTV^+^CD25^+^ and CD4^+^CTV^+^CD44^+^ populations, and histograms of CTV MFI dilution of different culture conditions (T:M-MDSCs 1:1; T:G-MDSCs 3:1). Histogram plots were gated on CD4^+^CTV^+^ cells and thus represent Teff cell proliferation. Numbers in histograms represent percentages of proliferated cells. Representative data from two independent mouse samples (*n*) are shown. Illustration created with BioRender.com.

To provide evidence for the functional re-programming of MDSC subsets in *Nlrp3*-deficient mice as suggested by the transcriptomic analysis, we set up an *in vitro* suppression assay. To this end, highly pure M-MDSCs and G-MDSCs were isolated from the spleens of melanoma-bearing *Nlrp3^-/-^* and WT animals and co-cultured with CellTrace Violet (CTV)-labeled T effector (CD4^+^Foxp3^-^) cells sorted from naïve Foxp3^GFP^ mice in the presence of anti-CD3/anti-CD28 activation beads (**Figure 7A**). Both MDSC subsets from *Nlrp3^-/-^* mice displayed reduced suppressive ability compared with their WT counterparts as evidenced by decreased CTV dilution (**Figure 7B**). Furthermore, absence of T-cell suppression by *Nlrp3^-/-^* MDSC subsets was accompanied by enhanced activation of T cells as demonstrated by the CD25 and CD44 expression (**Figure 7B**). Overall, these data reveal that NLRP3 deficiency attenuates the suppressive activity of MDSC subsets.

### Therapeutic targeting of NLRP3 inflammasome attenuates tumor growth and re-arranges the MDSC compartment

Considering that immunotherapy has demonstrated impressive results, yet in a small proportion of cancer patients, we sought to investigate whether pharmacologic inhibition of NLRP3 inflammasome in combination with ICI immunotherapy may demonstrate a synergistic therapeutic effect in tumor-bearing animals. For this reason, we first examined whether the MCC950 inhibitor, which prevents ATP hydrolysis, thus preserving an inactive conformation of NLRP3 inflammasome (39, 40), could efficiently inhibit NLRP3 inflammasome in primary splenocytes from naïve mice. Indeed, treatment with MCC950 of LPS-exposed splenocytes demonstrated a significant reduction of IL-1β secretion in culture supernatants confirming the potency to inhibit NLRP3 inflammasome (**Supplementary Figure 7B**). In the meantime, MCC950 did not demonstrate cytotoxic effects since assessment of 7-AAD expression by flow cytometry showed no differences between treated and non-treated cells (**Supplementary Figure 7A**).

**Figure 8:**
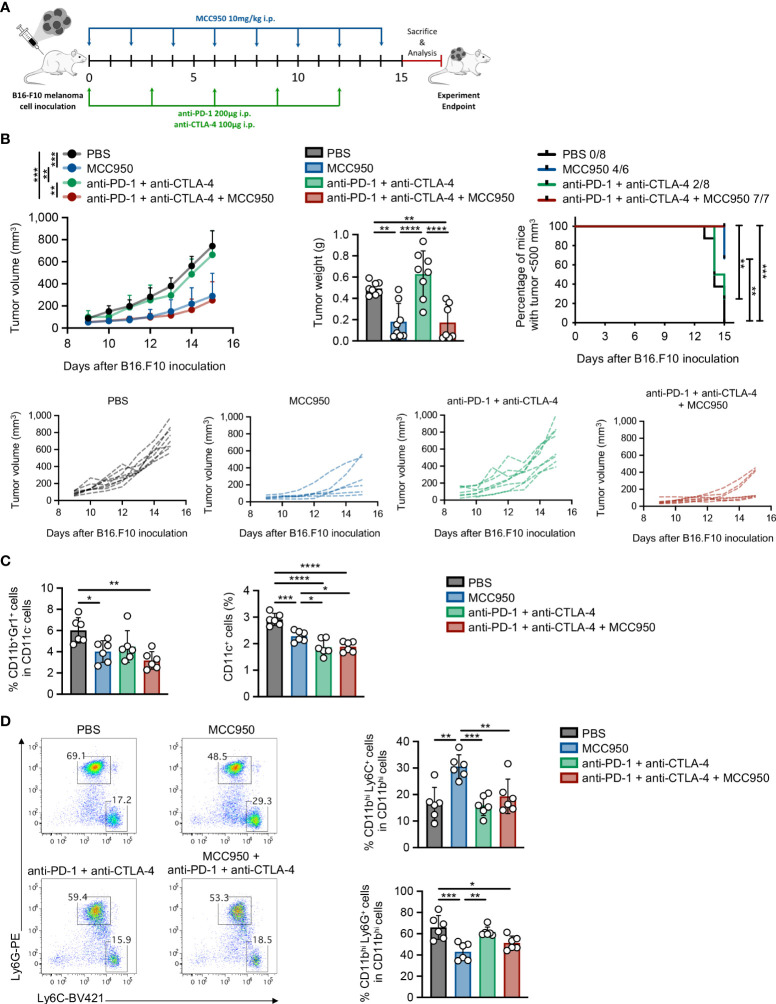
Pharmacologic inhibition of NLRP3 attenuates tumor growth and re-programs the MDSC compartment. **(A)** Experimental protocol for immunotherapy administration. B16.F10 melanoma-bearing C57BL/6J mice were treated with MCC950, anti-PD-1, and anti-CTLA-4 immune checkpoint inhibitors, or PBS as shown. Analysis was performed 15 days after tumor induction. **(B)** Tumor volume curve 9–15 days after B16.F10 inoculation, tumor weight on the experiment endpoint, and percentage of mice bearing tumors<500 mm^3^, with the ratio showing the number of mice with tumor<500 mm^3^/total injected mice (PBS: *n* = 8; MCC950: *n* = 8; anti-PD-1/anti-CTLA-4: *n* = 8; MCC950 + anti-PD-1/anti-CTLA-4: *n* = 7). **(C)** Frequencies of MDSCs and DCs in spleen tissue of B16.F10 melanoma-bearing C57BL/6J mice treated with the indicated treatments (PBS: *n* = 6; MCC950: *n* = 6; anti-PD-1/anti-CTLA-4: *n* = 6; MCC950 + anti-PD-1/anti-CTLA-4: *n* = 6), determined by flow cytometric analysis. **(D)** Representative FACS plots and frequencies of spleen-infiltrating M-MDSCs and G-MDSCs subsets of B16.F10 melanoma-bearing C57BL/6J mice treated with the indicated treatments (PBS: *n* = 6; MCC950: *n* = 6; anti-PD-1/anti-CTLA-4: *n* = 6; MCC950 + anti-PD-1/anti-CTLA-4: *n* = 6), determined by flow cytometric analysis. Data are shown as mean ( ± S.D.). Data from one **(C, D)** and representative data from two **(B)** independent experiments are shown. Statistical significance was obtained by one-way ANOVA with Tukey’s multiple comparison test or log(rank) test **(B)**. Symbols: (*), *p* ≤ 0.05; (**), *p* ≤ 0.01; (***), *p* ≤ 0.001; (****), *p* ≤ 0.0001, *n* = biologically independent mouse samples.

In order to examine the therapeutic efficacy of MCC950 *in vivo* and whether it may possess a synergistic effect if combined with immune checkpoint immunotherapy, WT animals were inoculated with B16.F10 melanoma cells and treated with MCC950 alone or in combination with anti-PD-1 and anti-CTLA-4 monoclonal antibodies (**Figure 8A**). Strikingly, systemic pharmacological inhibition of NLRP3 inflammasome significantly reduced tumor growth in tumor-bearing compared to control-treated mice (**Figure 8B**). However, no significant differences in tumor growth were observed between MCC950-treated mice and those that received the combination treatment protocol (MCC950 + anti-PD-1/anti-CTLA-4 ICIs) (**Figure 8B**). The therapeutic effect of MCC950 was accompanied by modulation of the myeloid compartment and specifically a significant reduction of both MDSCs and CD11c^+^ DCs was observed (**Figure 8C**). Of interest, DC frequencies were further reduced in mice treated with the combination of MCC950 inhibitor and anti-PD-1/anti-CTLA-4 antibodies compared to MCC950 alone (**Figure 8C**). Importantly, pharmacologic MCC950-mediated inflammasome inhibition resulted in a significant increase in the spleen M-MDSCs and subsequent reduction of G-MDSCs as compared to the control-treated group (**Figure 8D**), recapitulating the results obtained in *Nlrp3^-/-^* tumor-inoculated animals. Collectively, therapeutic targeting of NLPR3 in tumor-bearing mice results in tumor regression and re-programming of the MDSC compartment, while no synergistic effect in combination with ICIs was observed.

## Discussion

Inflammation is considered one of the most important hallmarks of cancer. However, as of today, therapeutic attempts in targeting the various inflammatory mediators that abundantly presented in the TME has shown limited success, highlighting the existence of unappreciated mechanisms of pro-tumorigenic inflammation. Herein, we reveal that targeting of the NLRP3 inflammasome in host cells, promotes tumor regression and induces re-wiring of the MDSCs, which constitute a major mechanism of tumor immune evasion. Specifically, we report a phenotypic, transcriptomic and functional re-programming between the monocytic and granulocytic subsets of MDSCs, upon *Nlrp3* deletion, during tumor development. Finally, pharmacologic inhibition of inflammasome, attenuates tumor growth and re-programs the MDSC compartment in a similar fashion to genetic silencing of *Nlrp3*.

Our findings are consistent with a pro-tumorigenic role of NLRP3 expression. This is in agreement with several studies, demonstrating that NLRP3 activation promoted tumor development in experimental models (41–47) and is associated with susceptibility to melanoma (48) and with poor survival in patients with advanced colorectal cancer (49) or development of various hematologic malignancies (47, 50). Despite these findings, an anti-tumorigenic role of NLRP3 has also been described as for example in NLRP3-deficient animals that showed increased susceptibility to colitis-associated cancer induced by dextran sulfate sodium (DSS) (51). In this line, a single-nucleotide polymorphism in *Nlrp3* gene Q705K (rs35829419) was correlated with decreased survival in colorectal cancer patients (52) and was also mapped at high frequency in patients with pancreatic cancer (53). Our TCGA analysis also revealed a contrasting correlation of NLRP3 expression and survival in patients with melanoma and lung cancer, building upon the proposed dual role of NLRP3 in cancer. In line with this, a previous pan-cancer analysis of NLRP3-related genes revealed that 15 types of cancer out of the 24 types analyzed demonstrated differential expression of NLRP3 signatures compared to normal samples and also showed that NLRP3 score could serve as an independent prognostic factor in SKCM (24). What determines the pro- versus the anti-tumorigenic role of NLRP3 remains obscure. It is possible that in the inflammatory context of the TME, the cell type in which NLRP3 operates, the genetic background, and the tumor cell per se possess a decisive role on NLRP3 activation and function during tumor development. Accordingly, activation of NLRP3 in cancer-associated fibroblasts (CAFs) (43) or macrophages (44) promoted tumor growth and metastasis supporting its pro-tumorigenic role, whereas sensing of dying tumors by DCs led to activation of NLRP3 followed by IL-1β secretion, which showed to be required for priming of tumor-specific IFN-γ-producing cytotoxic T lymphocytes promoting anti-tumor immunity consistent with an anti-tumorigenic role of NLRP3 in this cell subset (21). Finally, targeting NLRP3 expression in B16.F10 melanoma cells attenuated tumor growth and limited the expansion of MDSCs (54). Of note, in the latter study, *Nlrp3^-/-^* did not exhibit significant differences regarding B16.F10 melanoma cell growth compared to WT animals, in contrast to what we report. This discrepancy may account for the different strains of *Nlrp3^-/-^* mice used and/or the different melanoma cell numbers and method of injections (mixing with Matrigel) used in the two studies. Although studies with conditional targeting of *Nlrp3* in diverse cell types of the TME are still lacking, understanding the inflammasome role in an individual cell population that orchestrates the tumor immune evasion processes will provide further insights into the functional importance of this pathway and may introduce novel therapeutic targets.

Accumulating evidence in both preclinical models and patients with malignancies have established an important role of MDSCs in impeding tumor immune surveillance (5, 6, 8). Furthermore, MDSC-mediated suppression is an important mechanism of immunotherapy resistance. Therefore, major efforts over the last decade were placed for MDSC targeting to enhance anti-tumor immunity and immunotherapy responses (8, 55, 56). However, the heterogeneous nature of MDSCs and the limited knowledge on mechanisms *via* which may exert their function in the diverse tumor settings prevented such attempts. Our results demonstrate that the NLRP3/IL-1β pathway is activated in MDSCs in melanoma-bearing animals. Importantly, NLRP3 deficiency shifted the balance between M- and G-MDSCs frequencies, and transcriptomic analysis revealed that in the absence of *Nlrp3*, both MDSC subsets exhibited a robust re-programming highlighted by enrichment in type I IFN signature, inflammatory pathway signaling antigen processing and presentation transcripts, which has been closely linked to the functional properties of MDSCs. In addition, M-MDSCs showed enrichment in mTOR signaling, antigen processing, and presentation transcripts, which is in line with the findings by Alissafi et al. in which autophagy-deficient M-MDSCs upregulated the antigen presentation machinery and MHC-II expression promoting anti-tumor immunity (9). Moreover, G-MDSCs from *Nlrp3^-/-^* mice not only downregulated *Lox1* and *Pdl1* expression, which are associated with G-MDSC identity (34) and tumor immune evasion (35, 36), respectively, but also demonstrated enrichment in type I IFN signatures, which is linked to re-programming of granulocytic cells in the TME (32). Furthermore, the differential expression analysis from our RNA-seq data did not reveal any difference in the gene expression of Arginase-1 (Arg-1) and nitric oxide synthase 2 (Nos2) immunosuppressive factors, in both MDSC subsets. However, *Nlrp3* deficiency downregulated the expression of Stat-1 and hypoxia-inducible factor (Hif) 1a in G-MDSCs, which are transcription factors related to the immunosuppressive properties of MDSCs mainly by regulating *Arg-1* and *Nos2* expression (57–59). Notably, the transcriptomic re-wiring of MDSC subsets in *Nlrp3^-/-^* mice was accompanied by loss of function in both subsets as demonstrated by their inability to suppress T-cell responses *in vitro*.

So far, NLPR3 activation has been shown to be involved in the mobilization and expansion/activation of MDSCs through IL-1β secretion, while NLRP3 or IL-1β inhibition limited the MDSC presence in the TME (20). Moreover, the progression of melanoma was found to be associated with elevated concentrations of IL-1β as compared to patients with stable disease, and enrichment of circulating monocytic MDSCs significantly correlated with a decreased progression free survival of melanoma patients (60). Interestingly, our results show that IL-1β is not decreased in the TME of melanoma-bearing *Nlrp3^-/-^* animals (data not shown), indicating that NLRP3 ablation may promote tumor regression and MDSC subset re-programming *via* an IL-1β-independent mechanism. Thus, IL-1β may be secreted in an inflammasome-independent fashion (61) or other inflammasome products like IL-18 are likely to explain these findings. In support, IL-18 has been shown to enhance the immunosuppressive properties of M-MDSCs and to promote their accumulation in the TME (62). Alternatively, NLRP3 may exert cell-intrinsic signaling, which could instruct the differentiation of MDSCs not only in the periphery but also in the BM. Indeed, our results point to decreased frequencies of GMPs in the BM of *Nlrp3^-/-^* mice, which may be a result of the reduced inflammation due to tumor regression or could be explained by an intrinsic effect due to the absence of NLRP3, which may imprint on MDSC generation and subset differentiation. These hypotheses need to be thoroughly investigated considering that NLRP3 expression is established in human and mouse HSPCs and its involvement in hematopoiesis is emerging (63).

Several stimuli have been reported to activate NLRP3, ranging from pathogen-associated molecular patterns and DAMPs leading to NFkB activation, to stressogenic molecules and pathways such as reactive oxygen species, hypoxia, and extracellular ATP (17). Our results demonstrated that extracellular ATP induces the functional activation of NLRP3 in tumor-induced MDSCs *ex vivo*, accompanied by IL-1β release. The mechanisms, however, leading to NLRP3 activation in MDSCs during tumor development remain obscure. The TME is characterized by hypoxic conditions, which may drive NLRP3 activation in infiltrated cells including MDSCs. However, expression of the hypoxia-inducible transcription factor Hif1a is downregulated in G-MDSCs from *Nlrp3*-deficient mice. In addition, tumor dying cells release large amounts of ATP, which mediate NLRP3 activation (21). Consistent with this, the transcriptomic profile of *Nlrp3^-/-^* G-MDSCs has shown upregulation of *P2ry2* and *P2ry13* purinergic receptor genes. Furthermore, recently, Treg cells showed to release large amounts of ATP, upon apoptosis in the TME (64), which may, in turn, activate the inflammasome in other cells in close proximity like MDSCs. This may be envisioned as a resistance mechanism in which therapeutic targeting of one suppressive axis (i.e., Tregs) may empower a second one (i.e., MDSCs) to cope with tumor immune evasion. In support of this hypothesis chemotherapy-induced cathepsin B release demonstrated to activate NLRP3 inflammasome in MDSCs, curtailing anti-tumor immunity (65). In contrast, ATP released from tumor cells (21) or perforin released from CD8^+^ cytotoxic T lymphocytes (CTLs) (66) activated NLRP3 in antigen-presenting cells (APCs) and promoted immunity against tumors. Overall, identification of pathways that lead to NLRP3 activation in MDSCs may provide novel insights into the specific targeting of inflammasome and its products to these potent suppressive cells.

One of the mechanisms of acquired resistance to immunotherapy is the immunosuppressive circuit in the TME, which blunts the induction of anti-tumor immune responses. ICIs revolutionized cancer treatment but still a significant percentage of patients fail to respond (1). Whether inflammasome activation contributes to immunotherapy resistance and whether its targeting could augment therapeutic efficacy is not fully understood. Our data show that pharmacologic inhibition of inflammasome induces regression of melanoma growth and re-programs the MDSC compartment in a similar fashion to *Nlrp3*-deficient mice. Of interest, the combination of the NLRP3 inhibitor with anti-CTLA-4/anti-PD-1 did not demonstrate a significant synergistic effect in regard to tumor growth but only in the frequencies of MDSC subsets. The extent of inflammation involvement in the enhancement of immunotherapy efficacy is unexplored. In this line, pharmacologic targeting of inflammasome de-repression, through disruption of the transmembrane protein TMEM176b, augments the therapeutic efficacy of anti-CTLA4/anti-PD1 by unleashing inflammasome activation (67). The inflammatory context and the cell type where anti-CTLA-4/anti-PD-1-mediated NLRP3 activation takes place to instruct anti-tumor immunity remain to be determined.

In conclusion, our findings place inflammasome in the therapeutic quiver of cancer, while further efforts should be aimed at overcoming immunotherapy resistance in combinatorial regimens incorporating inflammasome inhibitors.

## Data availability statement

The datasets presented in this study can be found in online repositories. The names of the repository/repositories and accession number(s) can be found below: Gene Expression Omnibus (GEO accession number: GSE188973).

## Ethics statement

The animal study was reviewed and approved by Institutional Committee of Protocol Evaluation of the IMBB together with the Directorates of Agricultural Economy and Veterinary, Region of Crete, Greece (14/10/2020 Heraklion, Greece, protocol 234446).

## Author contributions

IP designed and performed experiments, analysed the data, generated the figures and wrote the manuscript. MG performed bioinformatic analysis of RNA-seq data and interpretation. LB generated and provided critical reagents. AK performed the TCGA survival analysis and edited the manuscript. AH performed experiments, analysed data and critically edited the manuscript. PV designed and supervised the study, performed the data analysis and wrote the manuscript. All authors contributed to the article and approved the submitted version.

## Acknowledgments

We thank all the members of the P. Verginis laboratory for helpful discussions. We thank Efrosyni Markaki for assisting with experiments, and Pavlos Alexakos and Hara Roumpaki for technical assistance on animal handling and maintenance, in the animal facility of the Biomedical Research Foundation of the Academy of Athens [BRFAA] and Institute of Molecular Biology and Biotechnology Institute [IMBB], respectively. We also thank Anastasia Apostolidou and Hara Vlata for technical assistance on flow cytometry and cell sorting at BRFAA and IMBB, respectively, and Vasiliki Theodorou and Emmanuel Dialynas for assistance with the transcriptomic analysis. This work was supported by grants from the Greek General Secretariat of Research and Technology Aristeia II 3468, the T2EDK-02288, MDS-TARGET to P.V., and the COST Action Mye-EUNITER (BM1404, http://www.mye-euniter.eu), as part of the European Union Framework Program Horizon 2020.

## Conflict of interest

The authors declare that the research was conducted in the absence of any commercial or financial relationships that could be construed as a potential conflict of interest.

## Publisher’s note

All claims expressed in this article are solely those of the authors and do not necessarily represent those of their affiliated organizations, or those of the publisher, the editors and the reviewers. Any product that may be evaluated in this article, or claim that may be made by its manufacturer, is not guaranteed or endorsed by the publisher.
