# Supplementary material for: Ablation of NLRP3 inflammasome rewires MDSC function and promotes tumor regression
Source: Front Immunol. 2022 Aug 10;13:889075. doi: 10.3389/fimmu.2022.889075 (PMC9407017; doi:10.3389/fimmu.2022.889075)
Supplement: Supplementary file 1 [file DataSheet_1.pdf]

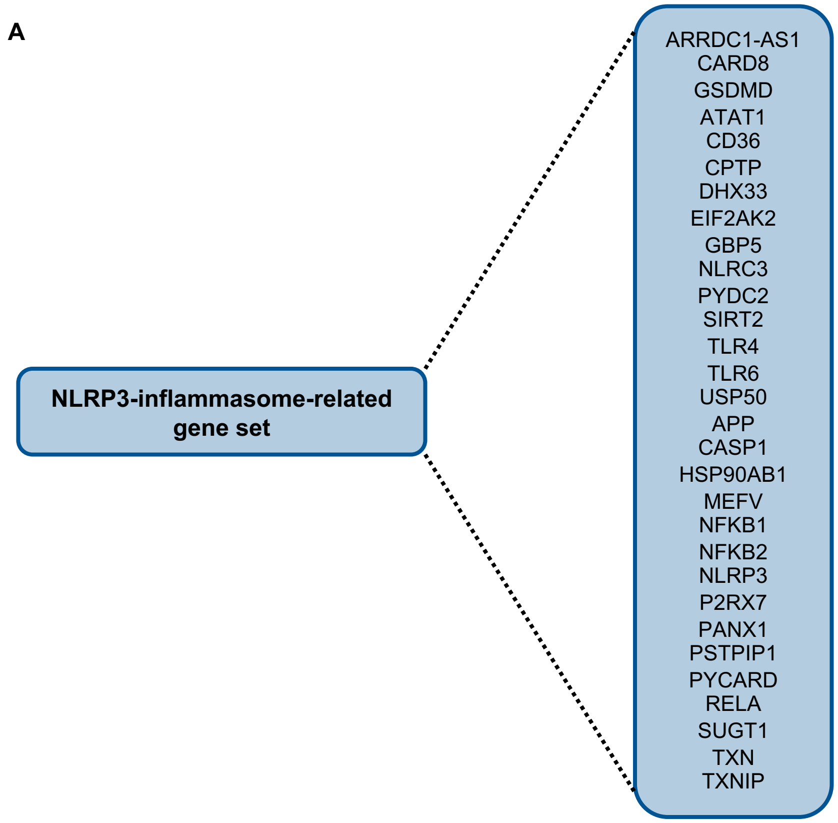

Supplementary Figure 1. NLRP3-inflammasome-related gene set. (A) Schematic representation showing the 30 pathway genes that were used in TCGA analysis to define the survival probability of LUSC and SKCM patients.

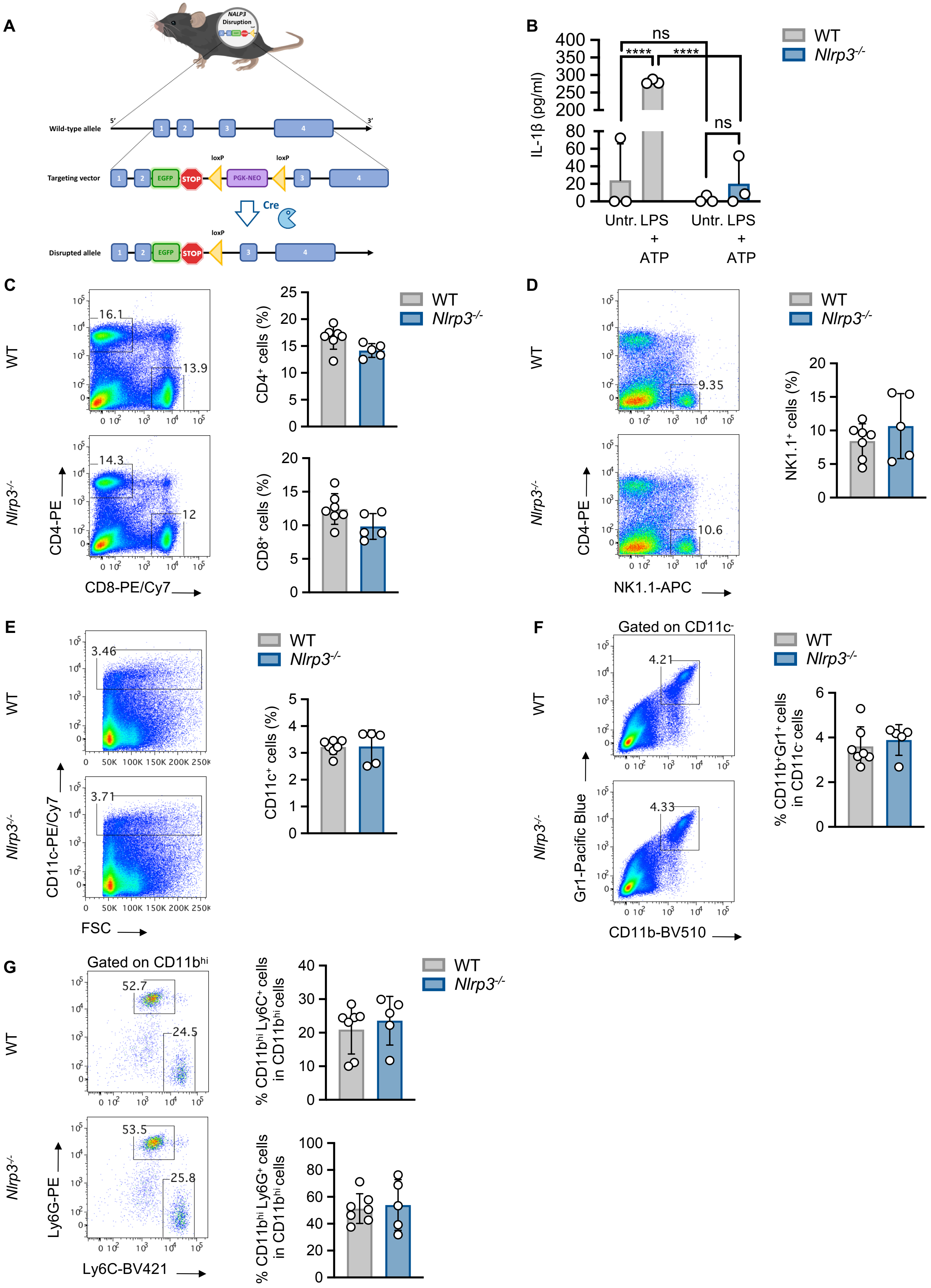
A
NALP3 Disruption
Wild-type allele
Targeting vector
EGFP
STOP
loxP
PGK-NEO
Cre
Disrupted allele
B
IL-1β (pg/ml)
ns
****
****
ns
Untr. LPS + ATP
Untr. LPS + ATP
WT
Nlrp3-/-
C
WT
Nlrp3-/-
16.1
13.9
14.3
12
CD4-PE
CD8-PE/Cy7
CD4+ cells (%)
CD8+ cells (%)
D
9.35
10.6
NK1.1-APC
NK1.1+ cells (%)
E
3.46
3.71
CD11c-PE/Cy7
FSC
CD11c+ cells (%)
F
Gated on CD11c-
4.21
4.33
Gr1-Pacific Blue
CD11b-BV510
% CD11b+Gr1+ cells in CD11c- cells
G
Gated on CD11bhi
52.7
24.5
53.5
25.8
Ly6G-PE
Ly6C-BV421
% CD11bhi Ly6C+ cells in CD11bhi cells
% CD11bhi Ly6G+ cells in CD11bhi cells

Supplementary Figure 2. *Nlrp3* deficiency does not affect immune homeostasis. (A) Schematic representation showing the gene targeting strategy for the generation of *Nlrp3* inflammasome knock out (*Nlrp3*$^{-/-}$) mouse model. **(B)** Quantification of IL-1β levels (pg ml^{-1}) in supernatants of untreated and LPS/ATP stimulated splenocytes from WT (n = 3) and *Nlrp3*$^{-/-}$ (n = 3) naïve mice, as determined by ELISA. **(C-G)** Representative fluorescence-activated cell sorting (FACS) plots and frequencies of spleen-infiltrating $CD4^+$ and $CD8^+$ T cells (**C**), NK1.1$^+$ cells (**D**), CD11c$^+$ dendritic cells (DCs) (**E**), CD11c$^-$CD11b$^+$Gr1$^+$ MDSC compartment (**F**) and CD11b^{high}Ly6C$^+$Ly6G$^-$ M-MDSCs and CD11b^{high}Ly6C$^-$Ly6G$^+$ G-MDSCs subsets (**G**) of naïve WT (n = 7) and *Nlrp3*$^{-/-}$ (n = 5) mice, determined by flow cytometric analysis. Data are shown as mean (±S.D.). Data from one **(B)** and representative data from two **(C-G)** independent experiments are shown. Statistical significance was obtained by one-way ANOVA with Tukey's multiple comparison test **(B)**. Symbols: (****), p≤0.0001, n = biologically independent mouse samples.

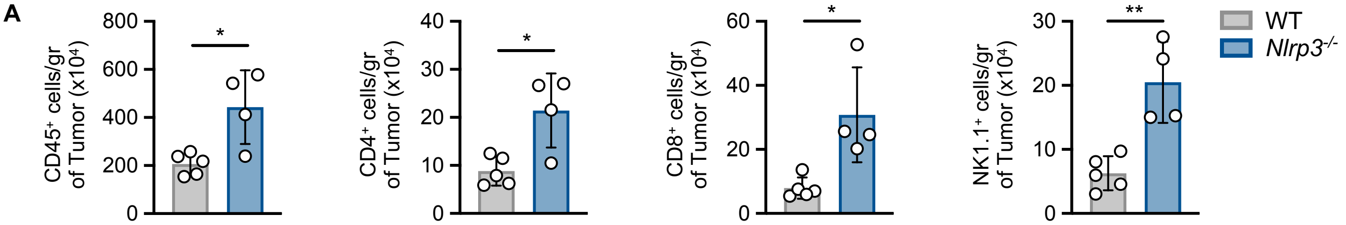

Supplementary Figure 3. *Nlrp3* deficiency modulates the anti-tumor immune response in TME. (A) Absolute numbers of tumor-infiltrating $CD45^+$, $CD4^+$, $CD8^+$ and NK1.1^+ cells per gram of tumor tissue in WT (n = 5) and *Nlrp3*$^{-/-}$ (n = 4) mice 15 days after B16.F10 inoculation. Data are shown as mean (±S.D.). Statistical significance was obtained by unpaired Student's t-test. Symbols: (*), p≤0.05; (**), p≤0.01, n = biologically independent mouse samples.

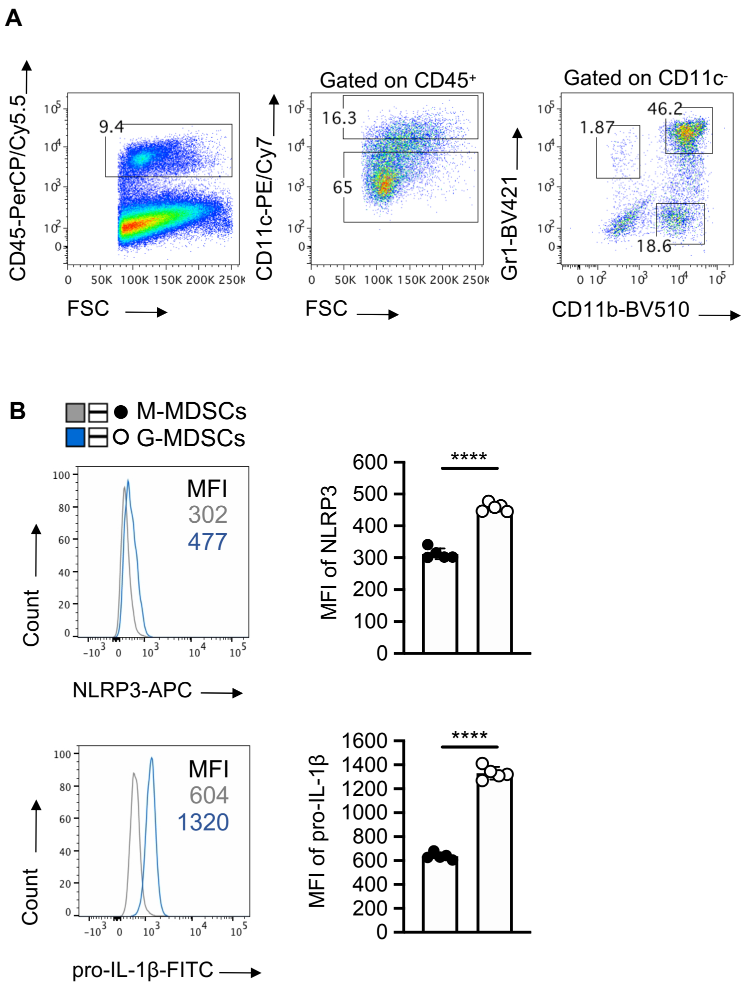

Supplementary Figure 4. Gating strategy and NLRP3 expression in MDSC subsets. (A, B) Data from tumor-bearing mice analysed 15 days after B16.F10 melanoma induction. **(A)** Gating strategy of $CD11c^{+}$, $CD11c^{-}CD11b^{+}Gr1^{+}$, $CD11c^{-}CD11b^{-}Gr1^{+}$ and $CD11c^{-}CD11b^{+}Gr1^{-}$ myeloid cell subsets in tumor site. **(B)** Representative histogram overlays of NLRP3 and pro-IL-1β expression and plots of NLRP3 and pro-IL-1β MFI of spleen-infiltrating M-MDSCs (n = 5) and G-MDSCs (n = 5) subsets. Data are shown as mean (±S.D.). Representative data from at least two independent experiments are shown. Statistical significance was obtained by unpaired Student's t-test. Symbols: (****), p≤0.0001, n = biologically independent mouse samples.

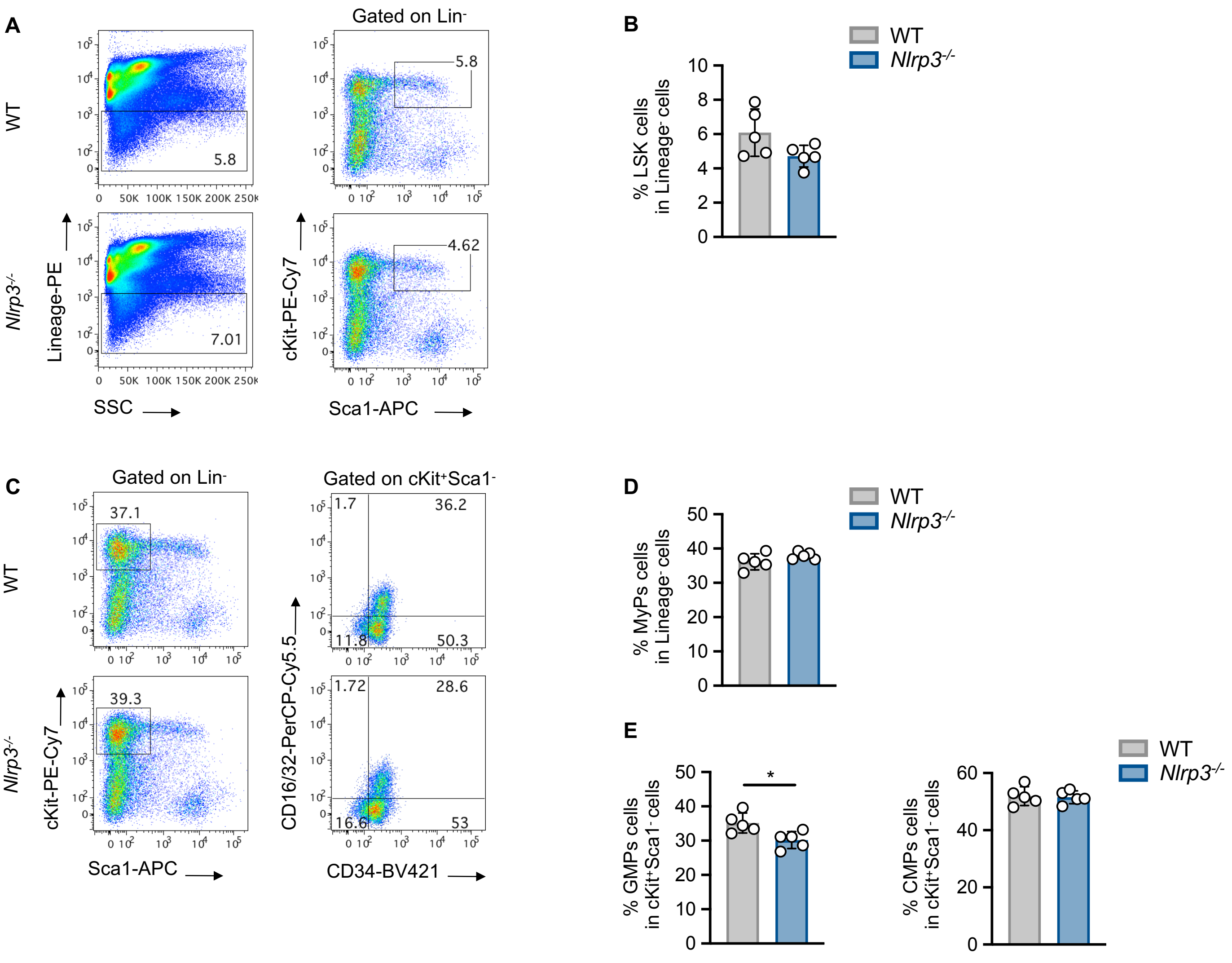

Supplementary Figure 5. Decreased frequencies of granulocyte-myeloid progenitors in the BM of *Nlrp3*$^{-/-}$ mice. (A, B) Gating strategy for the identification of the LSK compartment **(A)** and frequencies of LSK cells in Lineage^{-} (CD11b^{+}, CD16/32^{+}, Gr1^{+}, Ter119^{+}, B220^{+}, CD4^{+}, CD8^{+}) population **(B)** in the BM of WT (n = 5) and *Nlrp3*$^{-/-}$ (n = 5) mice 15 days after B16.F10 inoculation. **(C-E)** Gating strategy for the identification of MyPs subpopulations **(C)**, frequencies of MyPs in Lineage^{-} population **(D)** and frequencies of GMPs and CMPs within the MyP population **(E)** in the BM of WT (n = 5) and *Nlrp3*$^{-/-}$ (n = 5) mice 15 days after B16.F10 inoculation. Data are shown as mean (±S.D.). Representative data from three independent experiments are shown. Statistical significance was obtained by unpaired Student's t-test. Symbols: (*), p≤0.05, n = biologically independent mouse samples.

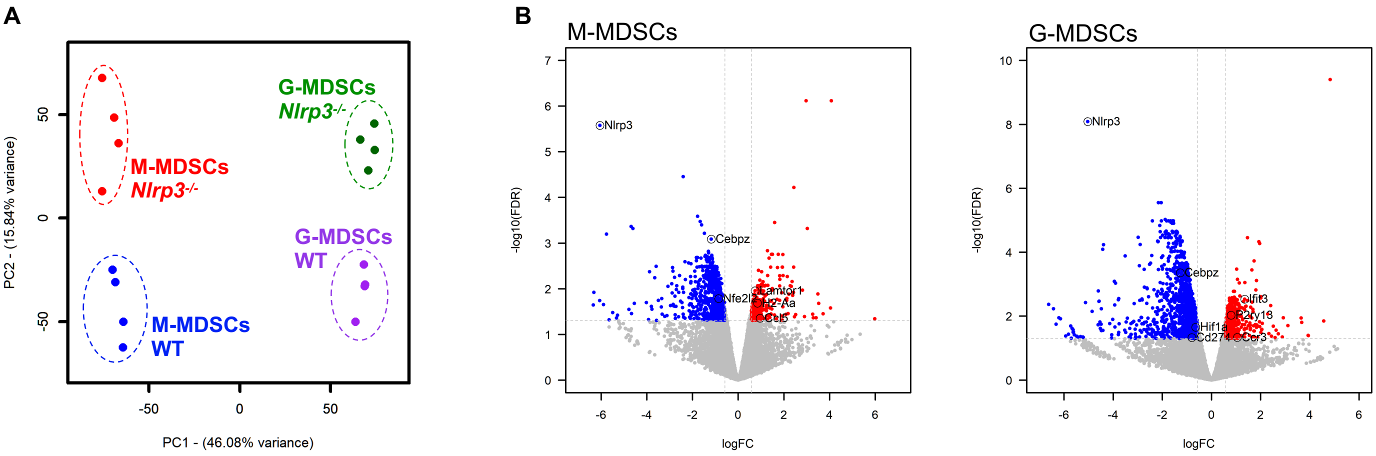

Supplementary Figure 6. Transcriptomic re-programming of M- and G-MDSCs from tumor-inoculated *Nlrp3*$^{-/-}$ mice. (A) Principal component analysis (PCA) showing the first 2 principal components (PC1, PC2) of RNAseq performed in G-/M-MDSCs from tumor-bearing mice of *Nlpr3*$^{-/-}$ and WT. The 4 groups are marked by different color as indicated in the legend alongside the plot. **(B)** Volcano plot of DEGs from M-MDSCs (left) and G-MDSCs (right) between *Nlrp3*$^{-/-}$ and WT tumor bearing mice. The blue dots denote downregulated genes and the red dots denote upregulated genes. Genes of interest have been noted in each volcano plot of both subsets.

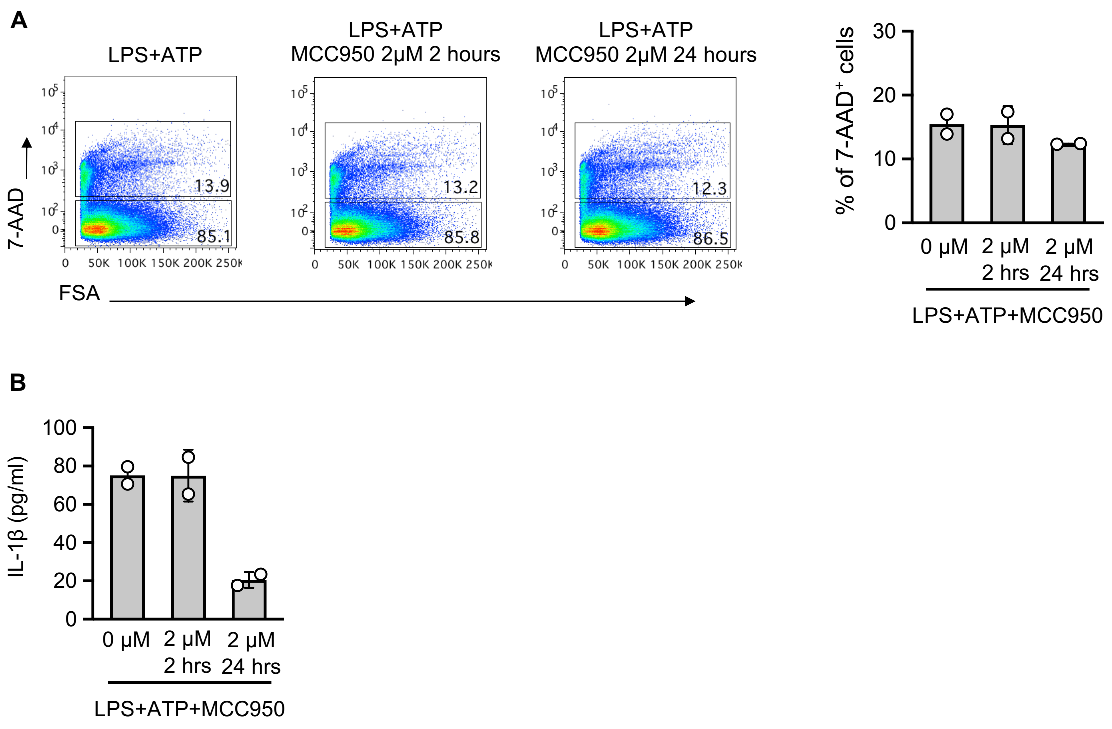

Supplementary Figure 7. MCC950 efficiently inhibits NLRP3 inflammasome activation. **(A)** Representative FACS plots and frequencies showing the percentages of 7-AAD$^+$ cells of WT splenocytes from naïve mice (n = 2), primed with LPS (1µg/ml), stimulated for NLRP3 inflammasome activity by ATP (5mM) inflammasome activator and treated with MCC950 (2µM) for the indicated time points. **(B)** Quantification of IL-1β levels (pg ml^{-1}) in the supernatants of splenocytes stimulated with LPS and ATP and treated with MCC950, as measured by ELISA.
